# Efficacy of Date Palm Pollen in the Male Sexual Dysfunction after Coronary Artery Bypass Graft: A Randomized, Double-Blind, Clinical Trial

**DOI:** 10.1155/2022/5032681

**Published:** 2022-03-28

**Authors:** Hamed Hooshang, Ali Vasheghani Farahani, Hossein Rezaeizadeh, Seyed Khalil Forouzannia, Farshid Alaeddini, Haleh Ashraf, Mehrdad Karimi

**Affiliations:** ^1^Department of Persian Medicine, School of Persian Medicine, Tehran University of Medical Sciences, Tehran, Iran; ^2^Clinical Cardiac Electrophysiology, Department of Cardiology, School of Medicine, Cardiac Primary Prevention Research Center, Cardiovascular Diseases Research Institute, Tehran Heart Center, University of Medical Sciences, Tehran, Iran; ^3^Traditional Medicine, Department of Persian Medicine, School of Persian Medicine, Cardiac Primary Prevention Research Center, Cardiovascular Diseases Research Institute, Tehran University of Medical Sciences, Tehran, Iran; ^4^Department of General Surgery, School of Medicine, Research Center for Advanced Technologies in Cardiovascular Medicine, Cardiovascular Diseases Research Institute, Tehran Heart Center, Tehran University of Medical Sciences, Tehran, Iran; ^5^Department of Research, Tehran Heart Center, Tehran University of Medical Sciences, Tehran, Iran; ^6^Cardiology Sina Hospital, Tehran University of Medical Sciences, Tehran, Iran; ^7^Traditional Medicine, Department of Persian Medicine, School of Persian Medicine, Tehran University of Medical Sciences, Tehran, Iran

## Abstract

**Background:**

Bypass graft surgery of the coronary artery has a significant effect on the creation and development of sexual dysfunction among male patients. The previous studies have demonstrated that date palm pollen (DPP) increases the count and quality of sperm. Additionally, it has been shown that DPP has a protective effect against myocardial infarction and cardiac remodeling. Therefore, this is the first study investigating the impact of DPP (*Phoenix dactylifera L.*) on managing male sexual dysfunction after coronary artery bypass graft.

**Methods:**

This randomized, double-blind, placebo-controlled clinical trial was conducted on 60 patients (DPP group *n* = 30, control group *n* = 30) of Iranian men after coronary artery bypass graft. Two parallel groups were randomly generated from the study participants. The intervention group was prescribed 3 grams of the powder of DPP twice a day (9 AM and 9 PM) for two months, while the control group received the same prescription of the placebo powder.

**Results:**

The DPP consumption significantly increased the International Index of Erectile Function (IIEF) (from 23.21 to 46.57) and the Hurlbert Index of Sexual Desire (HISD) (from 59.39 to 64.45) scores over time in the intervention group. However, there were no significant changes in the control group.

**Conclusion:**

Daily intake of 6 g DPP for two months exhibited beneficial effects on the symptoms of male sexual dysfunction in patients who have undergone coronary artery bypass graft (CABG).

## 1. Introduction

Sexual dysfunction represents a highly prevalent problem among male patients who undergo coronary artery bypass graft surgery (CABG). These patients involve in issues such as erectile dysfunction, reduced libido, and premature ejaculation [1]. It is estimated that sexual dysfunction affects roughly 10–25% of middle-aged and older men [2]. Previous studies showed that around three-quarters of cardiac disease patients experience some degree of sexual dysfunction [3]. As mentioned, this condition is common among patients who have undergone CABG [4], with some patients not being able to resume regular sexual activity [5]. Such patients have an impaired perception of well-being together with an attenuated quality of life. Hence, sexual function recovery fulfills a significant role in preserving the quality of life following cardiac operations [6].

The exact cause of sexual dysfunction is not fully understood [7]. Moreover, there are relatively limited treatments in reproductive medicine being directed at these issues of male health [8]. Currently, among those facing chronic conditions, there is a trend toward using herbal medicine and other forms of complementary and alternative medicine [9–12]. Although some traditional remedies and herbal drugs have been used by patients with sexual dysfunction, a significant proportion of medicinal plants have not yet been scientifically evaluated in this regard [13–15].

*Phoenix dactylifera* (date palm pollen, DPP) is the male reproductive dust of palm flowers used since ancient times as an aphrodisiac and fertility enhancer in patients with sexual dysfunction or infertility [16]. Experimental studies have revealed that DPP increases both sperm count and quality [17]. Moreover, previous studies demonstrated that DPP has a protective effect against myocardial infarction (via control of hyperlipidemia) and cardiac remodeling [18]. Phytochemical research has shown that the presence of flavonoids, sterol derivatives, and amino acids in the pollen may be responsible for its pharmacological effects [19]. Despite numerous clinical and animal studies on sexual dysfunction, no studies have examined the impact of DPP on male sexual dysfunction after coronary artery bypass grafting [20]. Hence, we evaluated DPP efficacy in managing male sexual dysfunction after CABG in this randomized, placebo-controlled trial.

## 2. Methods

### 2.1. Study Design

This study took the form of a randomized, double-blind, placebo-controlled clinical trial and was carried out in Tehran Heart Center in Tehran, Iran, from September 2019 to August 2020. We evaluated the efficacy of date palm pollen in the management of the symptoms of male sexual dysfunction after CABG. The study protocol was approved by the Ethics Committee of Tehran University of Medical Sciences (IR.TUMS.VCR.REC.1398.915) and is registered in the Iranian Registry of Clinical Trials (IRCT20191228045911N1). Informed consent was obtained from all participants; the entire study was conducted according to the 2013 revision of the Declaration of Helsinki.

### 2.2. Participants

The inclusion criteria for recruiting in this trial were as follows: men aged between 40 and 70 years who had undergone CABG, complained from sexual dysfunction, were willing to participate in the study, had an International Index of Erectile Function (IIEF) score of less than 50, and had a cardiac ejection fraction above 30%. Also, the exclusion criteria of this study were as follows: having uncontrolled thyroid disease, anemia, prostate cancer, renal or hepatic dysfunction, major depressive disorder, history of myocardial infarction in the last six months, history of cardiac valve replacement, history of sexual dysfunction before cardiac surgery, and history of allergy to pollen.

### 2.3. Sample Size Estimation

As there was no similar study, G∗power software (version 3.1.9) [21] was used to calculate the sample size. To achieve a large effect size (*d* = 0.8) with static power of 0.8 at a significant level of 0.05, a total of 26 patients were needed in each group. However, this was increased to 30 participants to consider a 10% probable dropout rate.

### 2.4. Randomization, Blinding, and Allocation Concealment

Sixty patients who presented at the Tehran Heart Center (Tehran, Iran) and met the inclusion criteria were subjected to prospective sequential sampling for random allocation into the intervention and control groups. Randomization was done by a biostatistician using block randomization in the Number Cruncher Statistical System (NCSS) software (size 4 per block). The drug and placebo powder sachets were labeled by the pharmacist using the same randomization list, with the identity of each being concealed until the end of the project. Patients, researchers, and medication deliverers were not aware of the group allocation. The placebo powder sachets were similar to the pollen powder sachets in terms of color, viscosity, and weight.

### 2.5. Preparation of Drug and Placebo

In early spring, palm pollen was collected from male date trees in Jahrom (Fars province, Iran). An herbal pharmacologist authenticated and deposited palm pollen in the herbarium of Jahrom University of Medical Sciences (specimen voucher number: 373846). The collected pollen was kept in a glass bottle and then discharged into sachets for administration.

The placebo powder was prepared by a pharmacist using standard Avicel powder, an inert substance with the same color as date palm pollen. This product is used as a fat replacer and is composed of microcrystalline cellulose that is partially hydrolyzed with acid before being reduced to a fine powder.

### 2.6. Intervention

Two parallel groups were randomly generated from the study participants. The intervention group was prescribed 3 grams of the powder of DPP twice a day (9 AM and 9 PM) for two months, while the control group received the same prescription of the placebo powder. All participants were asked not to take any other herbal and alternative medicine during the study.

### 2.7. Outcome Measures

The outcomes of the study were collected by a staff who was not aware of group assignments. A socio-demographic questionnaire was used to take the basic characteristics of the population such as age, body mass index, smoking, blood pressure, and lipid profile.

The Hurlbert Index of Sexual Desire (HISD) and IIEF scores were considered as the outcome measures of this study. The IIEF consists of 15 questions divided into five domains: erectile function, sexual desire, orgasmic function, intercourse satisfaction, and overall satisfaction. A score of 1 to 5 is given for each item, with a “0” option sometimes being available to denote the complete absence of sexual stimulation/intercourse [7]. Higher scores represent better sexual functioning. The HISD is composed of 25 questions graded from 0 to 4 on a Likert scale, with the total score ranging from 0 to 100. The lower one's score, the lower their sexual desire [9]. All outcomes were evaluated at the beginning, the first month, and the end of the study (second month).

The signs of an allergic reaction were explained to all participants; they were asked to report any potential side effects of the prescribed drugs immediately.

### 2.8. Statistical Methods

Initially, for comparison of baseline characteristics between groups, an independent *t*-test was used for continuous variables and was expressed as mean ± SD. A chi-square test was used for categorical variables and was presented as numbers. The mean scores of HISD and IIEF were compared between groups by an independent *t*-test. General linear model (GLM) repeated measures analysis of variance was performed to compare intervention and placebo across the 3-time points of the baseline, after a month, and the end of the study. Statistical significance was considered when *p* values were below 0.05. Data analysis was done using the Statistical Package for Social Sciences (version 15; SPSS Inc., Chicago, IL, USA).

## 3. Results

### 3.1. Study Flow

**Figure 1:**
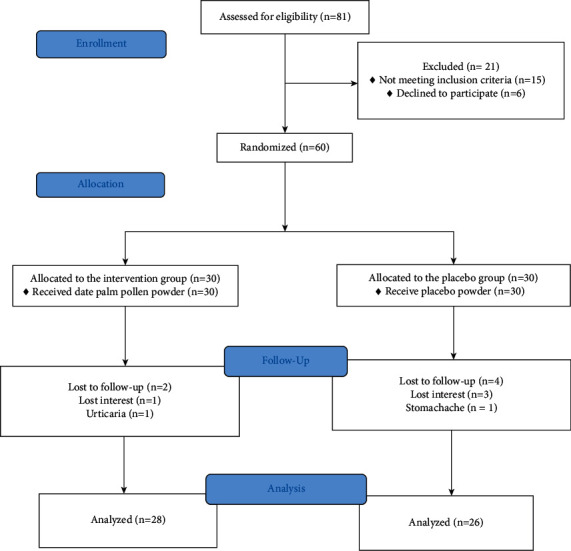
Flow diagram of the patients' enrolment, allocation, follow-up, and final analysis.

During the period between September 2019 and August 2020, we evaluated a total of 81 patients for eligibility. Of these, 15 were excluded because of not having eligibility criteria and 6 for unwillingness to take part. Finally, thirty patients were allocated to each of the control and intervention groups. At the end of the follow-up, two people from the DPP group have not completed the intervention due to lack of interest and urticaria, while four of the placebo recipients were excluded (due to stomachache and lack of interest). The study flow diagram is presented in Figure 1.

### 3.2. Baseline Data

The age BMI matching was performed to eliminate confounders' effect in DPP and control groups. Therefore, there was no significant difference between the two groups in terms of age (I: 61.93 ± 6.36 and C: 59.38 ± 8.91 years; *p* = 0.23) and BMI (I: 28.07 ± 4.30 and C: 27.00 ± 3.84; *p* = 0.398). Furthermore, the study groups were similar in terms of all baseline characteristics, except for the IIEF scores, which were significantly higher in the control group (I: 23.21 ± 14.08 and C: 32.31 ± 16.14; *p* = 0.03) (Table 1).

### 3.3. Clinical Response

**Figure 2:**
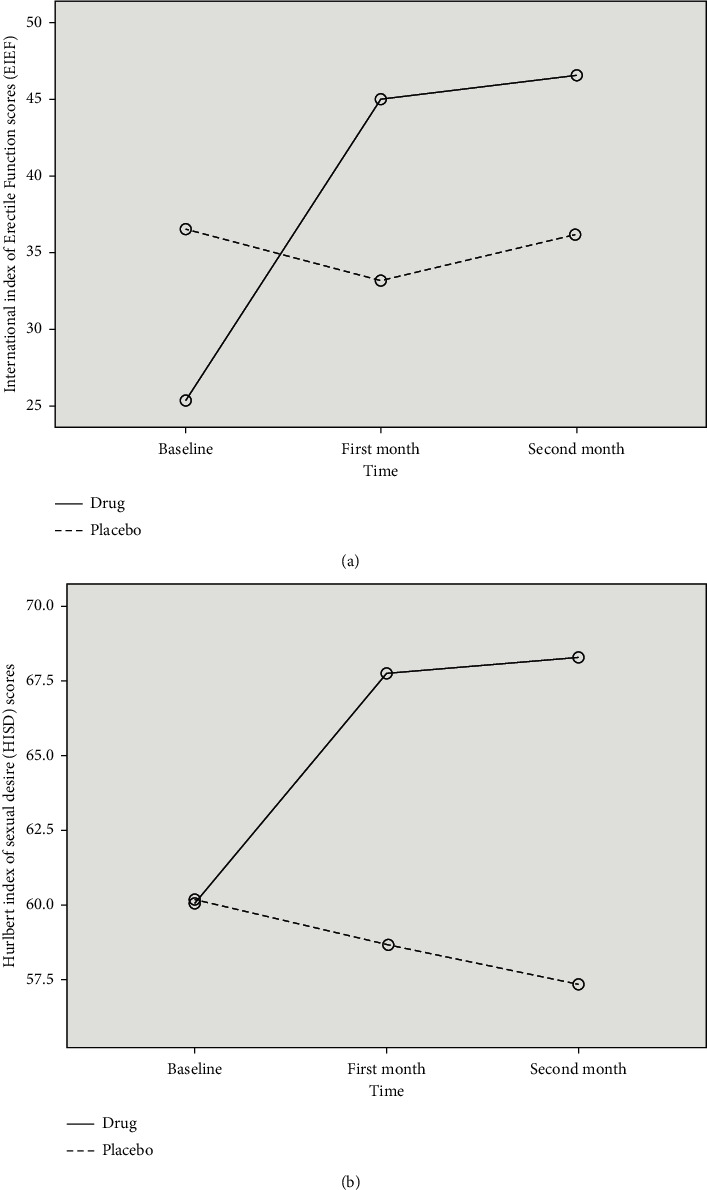
Changes in indices related to sexual function during the study period in each group.

In terms of changes within each group, we recorded a significant improvement in IIEF (from 23.21 to 46.57) and HISD (from 59.39 to 64.45) scores over time in the intervention group. However, this was not seen in the control group (IIEF: from 32.31 to 36.08; HISD: from 62.96 to 57.33). These changes are depicted in Figure 2. Through repeated measures analysis, we found that during the study period, the intervention led to a significant boost in both the IIEF and HISD scores (*p* = 0.001; *F* = 13.47 and *p* = 0.044; *F* = 4.10, respectively) when compared with the control (Figure 2).

## 4. Discussion

To our knowledge, this is the first clinical trial study in Iran and the world that examined the efficacy of date palm pollen in the management of male sexual dysfunction after coronary artery bypass graft. In our study, both libido and sexual function after DPP consumption improved significantly compared to a placebo. This study demonstrated that DPP leads to a significant increase in IIEF and HISD in males with sexual dysfunction.

The popularity of varied complementary and alternative medicine modalities, particularly herbal medicine in the prevention, treatment, or rehabilitation of patients with cardiovascular diseases, have been increased over recent decades [22, 23]. Coronary artery bypass graft surgery is one of the treatments of cardiovascular diseases [24]; however, it affects different aspects of patients' quality of life and leads to problems like sexual dysfunction, so more than half of these people experience disorders in sexual life [25]. Traditional Persian medicine (TPM) has introduced DPP as a safe and effective treatment to improve sexual satisfaction in males.

A number of trial and experimental studies indicated the benefits of this herbal product on male sexual performance. For example, as thyroid hormones have an important role in gonads' development and growth, a disorder in the thyroid gland can alter spermatogenesis [26]. El-Kashlan et al. did an investigation on male rats with thyroid dysfunction and concluded that DPP extract could neutralize adverse effects of fluctuation in the level of thyroid hormones and consequently improve fertility that may be due to its antioxidant profile. In addition, the benefits of DPP extract on the hypothalamic-pituitary-testicular axis maintained steroidogenesis that regulates testicular performance [27].

In line with the current study, Marbeen et al. also reported that DPP improved fertility rate in adult men with sexual dysfunction through a significant increase in serum level of testosterone, LH, and estradiol, number and motility of sperm, and diameter of seminiferous tubules [28]. Moreover, Bahmanpour et al. evaluated the effect of different doses of date palm gemmule on seven rats groups, which results presented sperm quality, spermatogenesis, and testis morphology promoted in groups treated with date palm gemmule [29].

An additional study by Mohamed and collaborators showed that the administration of DPP suspension solely or in combination with bee pollen in diabetic rats improved the disturbance in pituitary-testicular axis and testicular histology, which may be because of antiglycemic and antioxidant properties of these suspensions as well as promoted sexual desire [30]. Moreover, Baharara et al. demonstrated that exposure to electromagnetic fields induced significantly reduced sperm count, viability, and motility compared with controls, which intake of DPP could fight against these adverse effects [31].

The DPP extract possesses antitoxic properties plus antioxidant and anti-inflammatory effects. For instance, cadmium (Cd) is a toxic metal that can damage various organs, particularly the reproductive system [32]. Some animal studies demonstrated that treatment with DPP lowers the level of Cd in testis and consequently attenuates reproductive damage [33, 34]. In another study, date palm pit powder reversed spermatotoxicity induced by nicotine considerably [35].

Besides, Forouzannia et al. showed that sexual dysfunction increased significantly after CABG surgery due to a drop in sex hormone levels [36]. Hence, an increase in the level of sex hormones may be an effective approach in treating this disorder. Research by Shariati et al. concluded that date palm pit powder rose the level of testosterone and declined dihydrotestosterone level through suppressing 5-reductase enzyme as it contains some effective ingredients including palmitic, stearic, linoleic, and oleic acids [37]. Similarly, a study revealed that daily administration of 120 mg/kg DPP suspension promoted reproductive activity and testosterone level of serum and intratesticular [38]. Additionally, a trial in infertile men demonstrated that coadministration of DPP and zinc sulphate helped to promote serum level of LH, FSH, and testosterone hormones, count and motility of sperm, and sexual desire without any toxicity [39].

In addition, Daoud et al., in their animal study, revealed that DPP had a protective effect against myocardial infarction and isoproterenol-induced cardiac remodeling in rats through the inhibition of angiotensin-converting enzyme activity [18]. Hence, another probable mechanism of action of this herb in the improvement of sexual dysfunction following CABG may be related to its cardiotonic effect. Recently, erectile dysfunction is considered as a marker of vascular health. Nitric oxide (NO), which is released in the endothelium of vascular, has a pivotal role in the penile erection [40]. Date and its drives are rich sources of cardiovascular-protective components, such as polyphenols, potassium, magnesium, folate, selenium, fiber, and vitamin C. A molecular mechanism is claimed that polyphenols promote vasodilation via the generation of NO and inhibiting free radicals [41].

There are some strengths in this study including following the principles of clinical trials and using valid and reliable tools for outcome assessment; however, some limitations should be considered.

Firstly, low sample size was recruited, while it was computed with a standard formula. But, concerning the findings of the present study, it is proposed that large-scale studies should be conducted. Secondly, the lack of drug dose adjustment and dosing assessment represent critical limitations and should be taken into account by researchers in subsequent investigations. A short duration of follow-up is another drawback of the present work. Finally, some confounders, such as smoking and diabetes, can influence the efficacy of the intervention; nevertheless, we distributed patients with these conditions approximately equally in order to lower bias.

## 5. Conclusion

According to the results of this study, it seems that date palm pollen can, to some extent, relieve the symptoms of male sexual dysfunction in patients who have undergone CABG. However, further studies with larger sample sizes and longer follow-ups are needed to deepen our understanding of the efficacy and safety of this herbal product.

## Figures and Tables

**Table 1:** Baseline characteristics of patients in the study groups.

Variables	Date palm pollen (*n* = 30)	Placebo (*n* = 30)	*p* value
Age (years)	61.93 ± 6.36	59.38 ± 8.91	0.23
Body mass index (kg/m^2^)	28.07 ± 4.30	27.00 ± 3.84	0.39
Fasting blood sugar (mg/dL)	109.04 ± 21.53	106.50 ± 27.16	0.71
Smoker (yes/no)	15/13	13/13	0.74
Systolic blood pressure (mmHg)	113.61 ± 10.3	116.67 ± 12.4	0.05
Diastolic BP (mmHg)	75.97 ± 4.1	76.39 ± 5.8	0.33
Triglycerides (mg/dL)	174.13 ± 121.02	156.24 ± 64.12	0.54
LDL-cholesterol (mg/dL)	88.58 ± 34.10	91.10 ± 33.53	0.50
Cardiac ejection fraction (%)	44.60 ± 10.19	44.05 ± 8.30	0.84
International Index of Erectile Function score	23.21 ± 14.08	32.31 ± 16.14	0.03
Hurlbert Index of Sexual Desire score	59.39 ± 13.09	62.96 ± 12.50	0.31

Categorical variables are represented as a percentage (%) and continuous data are represented as mean ± SD.

## Data Availability

The data used to support this study are available from the corresponding author on reasonable request.
